# Some Recent Cases of Epidemic Cerebro-Spinal Meningitis

**Published:** 1913-06

**Authors:** J. R. Charles

**Affiliations:** Physician to the Bristol Royal Infirmary.


### SOME RECENT CASES OF EPIDEMIC CEREBRO-SPINAL MENINGITIS.

BY

J. R. CHARLES, M.A., M.D. Cantab., F.R.C.P.,
*Physician to the Bristol Royal Infirmary.*

THE following cases of meningococcic cerebro-spinal meningitis are described because there can be little doubt that at the present time there is a small epidemic of this disease in Bristol; and further, the symptoms and signs, especially in the early stages, are by no means easy to elucidate without the use of special methods, such as that of lumbar puncture.

In the three cases here described Flexner's serum has been used. In the first case it appeared to exert a marvellously beneficial effect, the patient being rapidly cured, even though he was admitted in a comatose condition. Whether this was the effect of the serum, or merely due to coincidence, it is difficult to say, especially as urotropin was also freely given, as it was in the other cases.

One patient was the wife of one of the others, but whether she derived her infection directly from her husband or from a common source it is impossible to say. She sat, however, close by his side during his last illness.

**Case 1.**—L. H. T., aged 16, a male, was admitted to the Bristol Royal Infirmary on November 23rd, 1912. He had suffered from a headache for a fortnight, and had given up his work two days before admission. During that day he became unconscious, and remained so until admission. He was then semi-conscious, and could be roused by a stimulus. He had extensive herpes labialis, was irritable, resented being touched, and lay curled up in bed. Temp. 97.8; pulse 70; resp. 24. His temperature during his stay in the Infirmary only reached 100 deg. F. on three occasions, and was as a rule either about normal or slightly subnormal. His pupils were equal and reacted to light, and on accommodation; there was definite optic neuritis in the right eye and some swelling of the left disc; no squint or nystagmus. The other cranial nerves were normal. No paresis of any of the muscles of the limbs could be detected. The deep reflexes were normal, and the plantars gave a flexor response on each side. There was very slight rigidity of the neck and a doubtful Kernig's sign. On the 25th a lumbar puncture was performed. The cerebro-spinal fluid was under increased pressure, and consisted in appearance of almost pure pus, in which the meningococcus was demonstrated by Professor Walker Hall. On this day there was more rigidity of the neck, Kernig's sign was present, and also a divergent strabismus. The mental condition remained as before. After an equivalent amount of cerebro-spinal fluid had been removed, 15 c.c. of Flexner's anti-meningococcic serum were injected into the cerebro-spinal canal. The following day the patient was much less drowsy and could answer questions. His squint had gone, and he complained of no headache. The other physical signs remained much the same, except that the plantar response was extensor on both sides. 15 c.c. more serum were given subcutaneously. Improvement continued, and he was much brighter mentally. The cerebro-spinal fluid was, however, still very turbid, though fewer organisms were present. Another 20 c.c. of serum were given intra-spinally. On the 27th improvement continued. The cerebro-spinal fluid was nearly clear, and 20 c.c. of serum were again given intra-spinally. On the 29th his mental condition appeared normal; Kernig's sign, Babinski's sign, the strabismus and head retraction had all disappeared, and the swelling of the discs was subsiding.

Following this he made a rapid and uninterrupted recovery, and was discharged cured on December 21st, when the only thing which could be detected as abnormal in his condition was a little patch of lymph round the right disc. He has been seen on several occasions since, and remains perfectly well. It should also be added that during the acute stage of the disease 10 grains of urotropin were given every four hours.

**Case 2.**—H. G., aged 45, a male, was admitted on April 17th, 1913, to the Bristol Royal Infirmary (under the care of Dr. Prowse, who has very kindly given permission for the publication of the case), complaining of a pain in his head, neck and legs. He had been ill for four days, the illness beginning with a shivering attack. He became unconscious on the evening of the 16th, and was unconscious on admission. He also had a crop of herpes labialis. His breathing was stertorous. Temp. 100.4; pulse 92; resp. 32. His urine was acid, 1025, and contained a trace of albumen and some sugar. There was no acetone or diacetic acid. Nothing abnormal was found in the heart or abdomen. In the lungs there was a little bronchitis at the bases. The nervous system was examined in detail. There was no optic neuritis, squint or nystagmus, and, in short, no evidence of any irritation or paralysis of any of the cranial nerves. The knee-jerks were normal, the plantar reflexes normal, and no evidence of any disease of the nervous system could be found, with the exception of a slight positive Kernig's sign in each side. A lumbar puncture was performed. The cerebro-spinal fluid was found to be purulent.

Professor Walker Hall's report on this was as follows:—
"Cells: polymorphonuclear 80 per cent., endothelials 15 per cent., lymphocytes 5 per cent. Culture: gram negative, intra-cellular diplococci (meningococci) found."

The patient was given urotropin gr. x every six hours, and 30 c.c. of Flexner's serum was injected intra-spinally, after a similar amount of cerebro-spinal fluid had been removed. The patient, however, became more deeply unconscious, and died on the morning of the 18th, *i.e.* the day after admission, without the occurrence of any further abnormal sign in his nervous system.

**Case 3.**—E. G., aged 36, female, wife of Case 2, was admitted to the Bristol Royal Infirmary on April 28th, 1913, with pain in her head. A week before admission she woke up with an attack of shivering, followed by vomiting. During the day she became seized with acute generalised headache. The headache, vomiting and shivering continued for four days, when she noticed sharp, stabbing pains in her back, which lasted about a minute at a time. These pains had disappeared on admission. On admission her face was covered with perspiration. Temp. 103; pulse 100; resp. 26. There was some stiffness in the muscles of the neck; the knee-jerks were slightly exaggerated, but there was no other sign of any abnormality in the nervous system. The fundi were perfectly normal, and there was no trace of any Kernig's sign. She could answer questions perfectly rationally, and gave a history of old chorea and of previous stiff necks, and stated that she was quite sure her neck on previous occasions had been as stiff as it was then. The heart was slightly enlarged, and at the apex a sharp first sound was heard, preceded by a presystolic murmur. Nothing abnormal was found in the lungs or abdomen. The diagnosis appeared to be between a rheumatic stiff neck and possibly cerebro-spinal meningitis. It was decided to administer salicylates, and if necessary perform a lumbar puncture. She was first given aspirin and then large doses of sodium salicylate. The temperature fell from 104 to 97.2, but there was no improvement in her condition, and on April 30th the temperature rose again. A lumbar puncture was therefore performed, when a quantity of milky fluid was obtained, in which the meningococcus was found by Professor Walker Hall, who also reported that there was no phagocytosis by leucocytes. The neck remained stiff, Kernig's sign remained absent, the fundi remained normal, and no fresh nervous sign developed. She was then put on urotropin, and 30 c.c. of Flexner's serum were injected intra-spinally. On May 1st there was a very slight blurring of the right disc, and an indefinite Kernig's sign appeared, tache cérébrale also being evident. The patient was rather drowsy, breathing was irregular and associated with a good deal of yawning; vomiting occurred five times. On May 2nd 30 c.c. of cerebro-spinal fluid were withdrawn, which was less turbid than before, and 30 c.c. of Flexner's serum were injected. She was still more drowsy, and the right disc was a little more blurred. Temp. 102.6; pulse 90; resp. 40. Other nervous signs as before. On May 7th improvement had continued, but she was still rather drowsy. The temperature for two days had not exceeded 99.4.

NOTE.—May 19. Progress has been uninterrupted. She now feels quite well, and has no abnormal physical sign in her nervous system.

